# Atopic Manifestations in Children Born Preterm: A Long-Term Observational Study

**DOI:** 10.3390/children8100843

**Published:** 2021-09-24

**Authors:** Federica Pagano, Maria Giulia Conti, Giovanni Boscarino, Chiara Pannucci, Lucia Dito, Daniela Regoli, Maria Di Chiara, Giuseppe Battaglia, Rita Prota, Bianca Cinicola, Anna Maria Zicari, Marina Aloi, Salvatore Oliva, Gianluca Terrin

**Affiliations:** 1 Department of Maternal and Child Health, Policlinico Umberto I Hospital, Sapienza University of Rome, 00161 Rome, Italy; federica.pagano22@gmail.com (F.P.); mariagiulia.conti@uniroma1.it (M.G.C.); giovanni.boscarino@yahoo.com (G.B.); chiara.pannucci@gmail.com (C.P.); lucia.dito@yahoo.it (L.D.); dani.regoli@virgilio.it (D.R.); maria.dichiara@uniroma1.it (M.D.C.); giuseppe.battaglia23@libero.it (G.B.); rita-prota@libero.it (R.P.); biancacinicola@gmail.com (B.C.); annamaria.zicari@uniroma1.it (A.M.Z.); marina.aloi@uniroma1.it (M.A.); salvatore.oliva@uniroma1.it (S.O.); 2 Department of Molecular Medicine, Sapienza University of Rome, 00185 Rome, Italy

**Keywords:** follow-up, atopic dermatitis, wheeze, food allergy, gestational diabetes, antibiotic, cow milk protein

## Abstract

(1) Background: Preterm birth exposes the infant to the known risk factors for atopic diseases. We aimed to study the neonatal risk factors and to describe the clinical manifestations of atopy, including the march of symptoms, in a cohort of preschool children born preterm. (2) Methods: We enrolled neonates with gestational age < 32 weeks or birth weight < 1500 g. We classified patients in cases and controls according to the presence of at least one atopic manifestation. (3) Results: We observed 72 cases and 93 controls. Multivariate models showed that the administration of more than one cycle of antibiotics (B 0.902, *p* = 0.026) and gestational diabetes (B 1.207, *p* = 0.035) influence the risk of atopy in babies born preterm. In addition, risk of atopic dermatitis was influenced by gestational age < 29 weeks (B −1.710, *p* = 0.025) and gestational diabetes (B 1.275, *p* = 0.027). The risk of wheeze was associated with familiarity for asthma (B 1.392, *p* = 0.022) and the administration of more than one cycle of antibiotics (B 0.969, *p* = 0.025). We observed a significant reduction in the rate of atopic manifestation after 2 years of life (33.9% vs. 23.8%, *p* < 0.05). (4) Conclusions: Modifiable (gestational diabetes, antibiotics use) and unmodifiable (familiarity for asthma) conditions influence the risk of atopy in babies born preterm. Extreme prematurity reduces the risk of atopic dermatitis. Preterm babies showed a peculiar atopic march.

## 1. Introduction

Over the last 40 years, the worldwide prevalence of allergic diseases has increased considerably [1]. Typically, atopy affects children of Western countries or with a Western lifestyle, but nowadays, as a consequence of world globalization, progressive urbanization and the improvement of socioeconomic conditions, it has become a major public health concern also for rural areas [2]. The “hygiene hypothesis”, supports that the excess “cleanliness” of our environments has led to the decline in the number of infectious stimuli that are necessary for the development of the immune system [3]. Recent findings support that not only pathogenic but also nonpathogenic microorganisms can serve as stimuli for the immune system, deterring the development of atopic responses [3]. Beyond a genetic predisposition, the early exposure to a large number of modifiable factors, including caesarian section, gut microbiome, antibiotic use, skin damage, breastfeeding and weight gain, may contribute to the development of atopic disorders [4,5]. In the last trimester of pregnancy, the fetal immune system adapts to tolerate maternal and self-antigens, while also preparing for postnatal immune defense [5,6]. Preterm birth, interrupting this “immunological imprinting” and leading the exposure to protective or harmful extrauterine factors such as microbiota and nutritional antigens, may have consequences for the development of immune diseases, such as atopy [5]. Despite this immunological status and despite that preterm birth exposes the infant to most of the aforementioned risk factors, there is no evidence of a higher prevalence of atopy in preterm compared to term-born infants [7].

Usually, the first clinical manifestation is atopic dermatitis (AD) (appearing between the second month and the second year of life) that commonly coexists with food allergy (FA), followed by asthma (in childhood) and finally allergic rhinitis (in adulthood). This progression of events and clinical manifestations is typically referred to as atopic march [8]. Of note, current literature on preterm newborns is still lacking in analyzing the general prevalence of all atopic manifestations and their evolution according to infant growth. Studies have focused on cutaneous, respiratory or gastrointestinal manifestations separately, with scarce information on the atopic march of children born preterm [9,10,11].

On the basis of these considerations, we designed a prospective study aiming at evaluating the risk factors and at describing the clinical manifestations of atopy, including the march of symptoms, in a cohort of preschool children born preterm.

## 2. Materials and Methods

In this observational study, we considered eligible newborns with gestational age (GA) < 32 weeks or body weight (BW) < 1500 g at birth, consecutively observed in Neonatal Intensive Care Unit (NICU) of Policlinico Umberto I, Sapienza University of Rome, from January 2015 to December 2019. We included all subjects with a follow-up of at least 1 year. We excluded subjects with major congenital malformations, inborn errors of metabolism, intestinal and extraintestinal congenital diseases, transfer to other hospital or death within the first 72 h of life or with incomplete clinical data [12,13,14,15,16,17].

The study was conducted according to the guidelines of the Declaration of Helsinki and Ethics Committee of Policlinico Umberto I, Sapienza University of Rome (5089, 13 September 2018). Informed written consent was obtained from the parents of each enrolled newborn.

Medical staff caring for the enrolled children was blinded to the study aims. Clinical data were collected by researchers not involved in clinical practice in a specific data form, deidentified and codified [18,19]. A statistician, blinded to the study design and aims, received a codified database and performed data analysis.

Protocol for nutritional management has been previously described [20,21,22]. Breastfeeding was started as soon as feasible after birth. Preterm formula was given only if human milk was not accessible or sufficient and donor breastmilk was not available during the study period [23].

Three researchers unaware of the study aims assigned a diagnosis of AD, wheeze and FA during the pediatric visit scheduled in the follow-up program, according to guidelines [24,25,26,27]. We defined as cases children with at least one atopic manifestation among AD, wheeze or with a diagnosis of FA and as controls those who did not show any signs or symptoms referred to atopic disease during the follow-up period of up to 5 years. To implement data collection regarding risk factors, family history of asthma or atopy, nutritional management, time of onset and outcomes of allergic symptoms, we administered an anamnestic questionnaire performed by a physician unaware of the study aims.

Statistical analysis was performed using Statistical Package for Social Science software version 25.0 (SPSS Inc, Chicago, IL, USA). We checked for normality using the Shapiro–Wilk test. The mean and 95% confidence interval summarized continuous variables, and number and percentage described categorial variables. We used χ^2^ test for categorical variables and *t*-test or Mann–Whitney or McNamara for paired and unpaired variables.

To evaluate the influence of covariates on atopic manifestations, we performed binary logistic regression analysis using the variables statistically significant in univariate analysis as covariates. We evaluated the rate of atopic disease independently of GA and age of diagnosis, performing a sensitivity analysis with newborns born before and after 29 weeks of postmenstrual age (PMA) and before and after 2 years of life. For statistical significance, we considered a *p* value < 0.05.

## 3. Results

**Figure 1:**
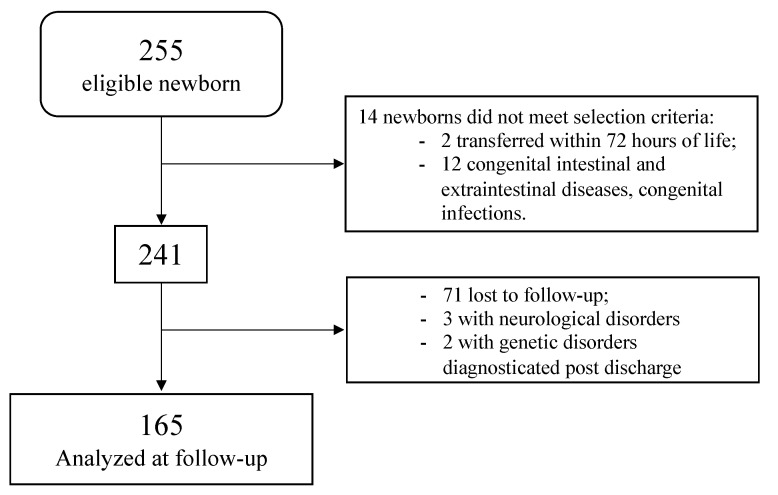
Flowchart.

As shown in Figure 1, of the 255 eligible newborns, we analyzed 165 infants at follow-up: 72 cases (43.6%) and 93 controls (56.3%).

Prenatal and perinatal clinical characteristics of enrolled case and control subjects are summarized in Table 1 and Table 2.

Newborns with at least one atopic manifestation and specifically with AD showed an increased rate of mothers with gestational diabetes compared with controls (Table 1). The GA at birth and male sex were significantly higher in AD cases than in controls (Table 2). Prevalence of familiarity for asthma was higher in children that developed wheeze compared with controls (Table 2).

In Table 3, we provide morbidity of enrolled cases and controls during hospitalization in the NICU. We observed that the cases of at least one atopic manifestation and the subgroup of subjects with wheeze received a prolonged administration of antibiotics in neonatal period and administration of more than one cycle of antibiotics (Table 3). In addition, the rate of children with infection requiring hospitalization is higher in the group of cases compared to controls (31.9% vs. 12.9%, *p* < 0.01), especially in wheeze subgroup (48.6% vs. 13.3%, *p* < 0.001). We observed one case of bronchiolitis.

Patients with FA introduced cow milk protein later (4 (0 to 9) days vs. 1 (0 to 1) day, *p* < 0.05).

**Figure 2:**
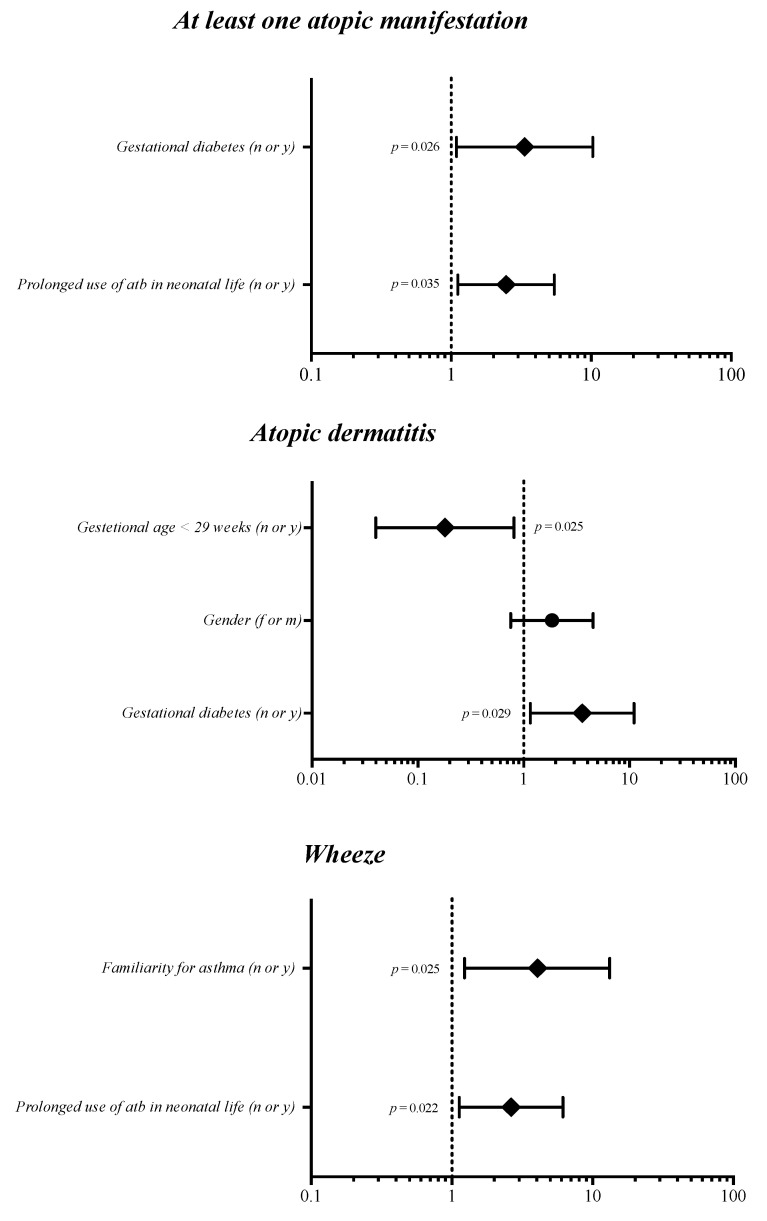
Multivariate analysis evaluating the influence of covariates on the risk of atopic manifestation. Notes: n or y, no or yes; f or m, female or male; atb, antibiotics. Prolonged use of antibiotics was defined as the administration of more than 1 cycle of antibiotics during the neonatal period.

Multivariate analysis showed that that the risk of developing at least one atopic manifestation in the first 5 years of life depends on the administration of more than one cycle of antibiotics and gestational diabetes (Figure 2). In addition, multivariate models showed that maternal gestational diabetes and GA > 29 were risk factors specific for AD, and familiarity for asthma and administration of more than one cycle of antibiotics in neonatal life influenced the occurrence of wheeze (Figure 2).

**Figure 3:**
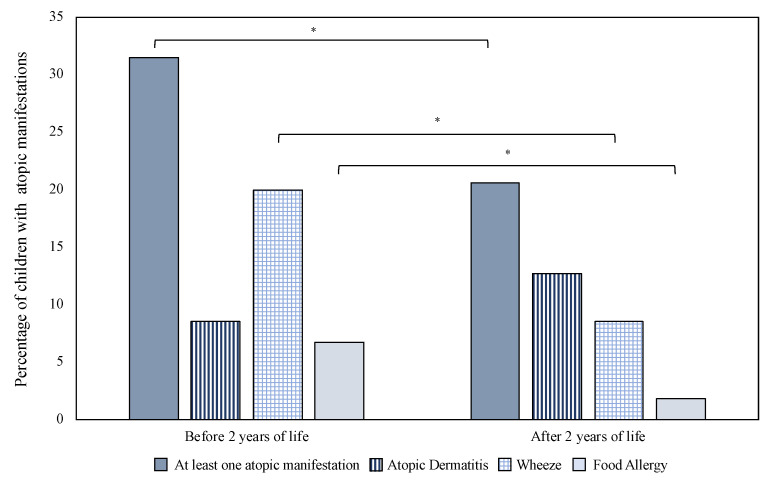
Atopic march in the study population. Notes: * *p* value < 0.05.

**Figure 4:**
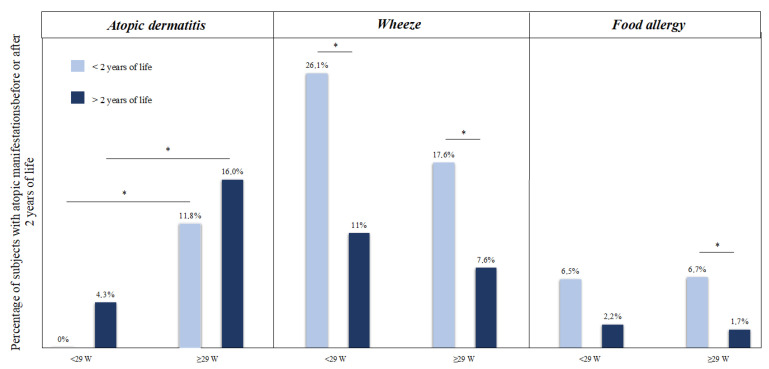
Atopic manifestation before and after 2 years of life in the study population. Notes: * *p* value < 0.05.

As shown in Figure 3, we observed a significant reduction in the rate of subjects with atopy starting from the second year of life. In particular, wheeze and FA decreased after 2 years of life, but AD increased after 2 years, although it did not reach statistical significance (Figure 3). This difference was observed also in the sensitivity model separately considering newborns born before and after 29 weeks of PMA (Figure 4). We observed a significant increase in the rate of patients born before 29 weeks of PMA with AD after 2 years of life compared with those born after 29 weeks of GA, and vice versa (Figure 4). Of note, none of the children born before 29 weeks of PMA presented signs of atopic dermatitis in the first 2 years of life (Figure 4).

## 4. Discussion

This observational study demonstrated that more than 40% of children born with GA < 32 weeks and body weight < 1500 g developed atopic manifestations during the first 5 years of life. If early administration of antibiotics increased the general risk of atopy, the risk of AD was associated also with maternal gestational diabetes and GA at birth, while the occurrence of wheeze was associated with familiarity for asthma.

Along with a reduced occurrence of atopic signs and symptoms with growing age, we observed also a peculiar atopic march consisting in a lower prevalence of respiratory and food allergy in children younger than 2 years compared with older children, associated with an increasing prevalence of atopic dermatitis after the first 2 years of life.

Previous studies investigating the general risk of atopy among prematurely born children gave conflicting results. Risk factors for atopic disease in this population are still undefined. In two different studies, Siltanel et al. demonstrated that prematurity is linked with a decreased long-term risk of atopy [7,28]. Discordance between the published data and our results may be explained by differences in study design. First, we included a population of very or extremely preterm infants, while Siltanen et al. compared term-born children and preterm ones, using a smaller sample size [7,28]. Furthermore, our study included, prospectively, patients admitted to the NICU in the last 5 years, whereas Siltanen et al. described, in a retrospective study, a population of children born in 1987–1988, thus probably receiving different neonatal standard of care [7,28]. Moreover, criteria to define the presence of atopy were structured to identify only IgE-mediated reactions, while we diagnosed atopic diseases referring to recent guidelines by an accurate clinical evaluation [24,25,27,29]. In a cross-sectional study, Ünal et al., enrolling 98 infants born before 37 weeks of GA, demonstrated that BPD is associated with reduced risk of general atopy [30]. This study did not provide information regarding extremely preterm infants, and the authors in this case also reported only IgE-mediated reactions at 2 years of life.

As occurred for general atopy, we observed that wheeze is significantly associated with the use of antibiotics in neonatal life. This statement probably depends on the large number of cases of wheeze in our population, which could have influenced results regarding the general risk of atopy. Similarly to our study, Carstens et al., in a secondary analysis of a randomized control trial selecting 142 infants born before 32 weeks of GA or VLBW, found that the use of antibiotics in neonatal life is a risk factor for recurrent wheeze [31]. In a previous study, an association between prematurity and wheeze was reported, but this result was obtained in a population of preterm infants born before 37 weeks during a follow-up period of only 2 years, without specific analysis for extremely premature babies [30].

Published data on the prevalence of AD in premature compared with term children are conflicting, partly due to differences in the study design. Some prospective studies analyzed the relative risk for premature children of having AD and found no significant differences between preterm and term children [32,33]. Other evidence suggests that prematurity and prematurity-related conditions are protective, as hospitalization due to AD becomes more common with increasing GA [34,35,36]. In agreement with studies including children born at term, we found that maternal gestational diabetes may increase the risk of AD [37]. The previous study of Kumar et al. [38] showed that maternal diabetes mellitus increased the risk of AD (RR: 1.72, 95%CI: 1.03–1.95) and early allergic sensitization (RR: 1.49, 95%CI: 0.96–1.65) in a large cohort of children born at term. Our multivariate analysis confirmed that maternal diabetes represented a risk factor for AD also in a population of children born preterm.

In our population, the late introduction of cow milk proteins was found to be a risk factor for FA. The Learning Early About Peanuts (LEAP) trial demonstrates how the late introduction of allergens increases the risk of food allergy [39]. More studies are necessary to clarify the role of the late introduction of cow milk proteins, though it is unfeasible to design randomized blinded trials on human milk or formula for obvious ethical reasons.

Differently from previously published studies, our study focused only on preterm newborns, with a specific analysis for extremely preterm neonates. There are many works that describe the atopic march and manifestation in healthy full-term newborns, but studies focused only on preterm newborns are lacking. In addition, we focused on clinical evaluation so our findings, if confirmed by future studies, could improve the clinical practice and decisions of neonatologists to prevent modifiable factors of atopy for this vulnerable population.

Hypotheses subtending our results are various, and although this is not the aim of the study, we tried to make some suggestions considering pathogenetic mechanisms best suited to the population. Dysbiosis of the gut flora, which frequently occurs after treatment with broad-spectrum antibiotics, may influence the development of immune response in early life [40]. An association between atopy and gut microbiota is established during infancy, representing a novel target for intervention [41].

Mothers with gestational diabetes have higher levels of TNF-alpha, leptin and visfatin and lower levels of adiponectin [42,43] that, in turn, might have a significant impact on the development of the immune response in infancy. However, future studies will need to be carried out to evaluate this specific aspect.

There is a growing body of evidence showing that early introduction of allergens might actually be beneficial in preventing food allergy and that delaying introduction might contribute to allergic disease [44,45]. The mechanism behind the development of tolerance to food proteins is complex; it requires a certain state of immune maturation and gut colonization. Exposure to food proteins during a critical window seems to be crucial in achieving tolerance. Our data suggest that this timeframe may be anticipated in the first months of life in children born preterm [46].

The atopic march is defined as a natural progression of allergic diseases mediated by Th2 lymphocytes. It generally starts early in infancy with AD and evolves to IgE-mediated FA, asthma and allergic rhinitis in later childhood [47]. This theory has been widely studied in cross-sectional and long-term longitudinal studies [48,49]. Among them, prospective birth cohort studies showed that early-onset AD is the main risk factor for other subsequent allergic diseases [50,51,52]. In our population, we observed a peculiar “atopic march” evolution of atopic symptoms during the first years of life, with an increasing rate of AD after the first 2 years of life, associated with a lowering prevalence of wheeze and FA, especially in patients born before 29 weeks of GA. In a prospective study including children aged 6–9 years, Barberio et al. found a “reverse” atopic march, paradoxically considering asthma as a risk factor for the later development of AD [53]. It is possible to hypothesize that early exposure to multiple risk factors of preterm neonates may influence the shift from Th2, which is a well-known hallmark of allergic disorders, to Th1 immune response [54]. However, further studies are necessary to shed light on this observation.

Despite being interesting, our findings should be interpreted taking into account some limitations. The main results may be related to the effects of chance (random error), bias or confounding factors. We verified that the rate of atopic manifestation persisted even after we corrected results for confounding variables statistically significant on univariate analysis. However, confounding variables still unknown or not considered in our analysis may have influenced the results. In univariate analysis, only cow milk protein introduction reached the statistical significance between cases and controls of FA, and thus we did not perform a multivariate model. To limit observer bias, the data for the analysis were collected by researchers who were not involved in the eligibility assessment, unaware of the study outcome and design and not involved in clinical practice. We discussed and defined a protocol for the collection, measurement and interpretation of data before starting the study. A third-party observer was involved to collect data on primary outcomes. Besides, a blinded statistician performed the data analysis. An additional limitation of the study is the lack of a control group of term-born infants, although there is a consistent body of literature reporting data on this population, while there are fewer studies on atopy and preterm infants. Finally, our observations were derived from a relatively small number of preterm newborns included in the study. However, this is a larger population of preterm infants born before 32 weeks of GA than those described in current literature. In addition, we reported the results of the analysis obtained in a population of extremely preterm newborns (<29 weeks of GA).

## 5. Conclusions

In conclusion, we identified some modifiable risk factors for atopy in children born preterm. Our results may have an impact on the clinical practice, suggesting some possible interventions: (1) to prevent and treat maternal gestational diabetes, (2) to limit the administration of antibiotics in neonatal life to when it is strictly necessary and (3) to not delay the introduction of cow milk proteins and/or other potentially allergenic foods. Future studies are advocated to clarify the mechanism of development and evolution of atopic diseases in this particular population.

## Figures and Tables

**Table 1:** Prenatal characteristics of the study population.

	All Newborns (n = 165)	At Least One Atopic Manifestation		Atopic Dermatitis		Wheeze		Food Allergy	
		Case (n = 72)	Control (n = 93)	Case (n = 32)	Control (n = 133)	Case (n = 37)	Control (n = 128)	Case (n = 12)	Control (n = 153)
Antenatal corticosteroids ^a^	112 (67.9)	46 (63.9)	66 (71.0)	20 (62.5)	92 (69.2)	24 (64.9)	88 (68.8)	7 (58.3)	105 (68.6)
Prepartum antibiotic prophylaxis ^b^	45 (27.4)	22 (31.0)	23 (24.7)	5 (15.6)	40 (30.1)	12 (32.4)	33 (26.0)	2 (16.7)	40 (26.1)
Gestational diabetes	16 (9.8)	11 (15.5) *	5 (5.4)	8 (25.0) **	8 (6.0)	4 (10.8)	12 (9.5)	1 (8.3)	15 (9.8)
Intrauterine growth restriction	17 (10.4)	7 (9.9)	10 (10.9)	5 (15.6)	12 (9.0)	1 (2.7)	16 (12.5)	2 (16.7)	15 (9.8)
Cesarean section	147 (89.1)	63 (87.5)	84 (90.3)	27 (84.4)	120 (90.2)	34 (91.9)	113 (88.3)	10 (83.3)	137 (89.5)
Maternal smoke during pregnancy	52 (31.5)	19 (26.4)	33 (35.5)	9 (28.1)	43 (32.3)	8 (21.6)	44 (34.4)	4 (33.3)	48 (31.4)
Familiarity for atopy	44 (26.7)	15 (20.8)	29 (31.2)	7 (21.9)	37 (27.8)	7 (18.9)	37 (28.9)	4 (33.3)	40 (26.1)
Familiarity for asthma	13 (7.9)	7 (9.7)	6 (6.5)	3 (9.4)	10 (7.5)	6 (16.2) *	7 (5.5)	0 (0)	13 (8.5)

Table legend: ^a^ Intramuscular steroid cycle in two doses of 12 mg over a 24 h period; ^b^ Penicillin or ampicillin or cefazoline or clindamycin every 4 h after the delivery; * *p* value < 0.05; ** *p* value < 0.01. Data are expressed as No. (%).

**Table 2:** Perinatal characteristics of the study population.

	All Newborns (n = 165)	At Least One Atopic Manifestation		Atopic Dermatitis		Wheeze		Food Allergy	
		Case (n = 72)	Control (n = 93)	Case (n = 32)	Control (n = 133)	Case (n = 37)	Control (n = 128)	Case (n = 12)	Control (n = 153)
Born in autumn or winter, *No. (%)*	66 (40.0)	42 (58.3)	57 (61.3)	13 (40.6)	53 (39.8)	18 (48.6)	48 (37.5)	3 (25.0)	63 (41.2)
Gestational age, *weeks*	30 (29 to 30)	30 (29 to 30)	30 (29 to 30)	31 (30 to 31) *	30 (29 to 30)	29 (29 to 30)	30 (29 to 30)	30 (28 to 32)	30 (29 to 30)
<29 weeks, *No. (%)*	46 (27.9)	16 (22.2)	30 (32.3)	2 (6.3) **	44 (33.1)	12 (32.4)	34 (26.6)	4 (33.3)	42 (27.5)
Birth weight, *grams*	1283 (1232 to 1333)	1325 (1255 to 1394)	1250 (1178 to 1322)	1359 (1258 to 1461)	1264 (1207 to 1322)	1289 (1188 to 1391)	1281 (1222 to 1340)	1280 (1089 to 1471)	1283 (1230 to 1336)
ELBW, *No. (%)*	37 (22.4)	14 (19.4)	23 (24.7)	6 (18.8)	31 (23.3)	7 (18.9)	30 (23.4)	3 (25.0)	34 (22.2)
SGA at birth, *No. (%)*	30 (18.2)	13 (18.1)	17 (18.3)	8 (25.0)	22 (16.5)	5 (13.5)	25 (19.5)	3 (25.0)	27 (17.6)
Male, *No. (%)*	86 (52.1)	43 (59.7)	43 (46.2)	22 (68.8) *	64 (48.1)	22 (59.5)	64 (50.0)	6 (50.0)	80 (52.3)
pH at birth	7.3 (7.2 to 7.3)	7.3 (7.2 to 7.3)	7.3 (7.2 to 7.3)	7.3 (7.2 to 7.3)	7.3 (7.2 to 7.3)	7.3 (7.2 to 7.3)	7.3 (7.2 to 7.3)	7.3 (7.2 to 7.3)	7.3 (7.2 to 7.3)
CRIB II score ^a^	6 (5 to 6)	6 (5 to 6)	6 (5 to 7)	5 (4 to 6)	6 (5 to 7)	6 (5 to 7)	6 (5 to 6)	5 (3 to 7)	6 (5 to 6)

Table legend: ^a^ CRIB II: clinical risk index for babies; SGA (Small for gestational age); * *p* value < 0.05; ** *p* value < 0.01. Data are expressed as mean (95% CI), when not specified.

**Table 3:** Morbidity characteristics of the study population during hospital stay and on follow-up.

	All Newborns (n = 165)	At Least One Atopic Manifestation		Atopic Dermatitis		Wheeze		Food Allergy	
		Case (n = 72)	Control (n = 93)	Case (n = 32)	Control (n = 133)	Case (n = 37)	Control (n = 128)	Case (n = 12)	Control (n = 153)
Necrotizing enterocolitis	6 (3.6)	5 (6.9)	1 (1.1)	3 (9.4)	3 (2.3)	1 (2.7)	5 (3.9)	1 (8.3)	5 (3.3)
Intraventricular hemorrhage	6 (3.7)	1 (1.4)	5 (5.4)	1 (3.1)	5 (3.8)	1 (2.7)	5 (4.1)	0 (0)	6 (3.9)
Bronchopulmonary dysplasia	7 (4.3)	2 (2.8)	5 (5.4)	1 (3.1)	6 (4.5)	2 (5.4)	5 (4.1)	0 (0)	7 (4.6)
Retinopathy of prematurity	21 (12.7)	11 (15.3)	20 (21.5)	2 (6.3)	19 (14.3)	4 (10.8)	17 (13.3)	1 (8.3)	20 (13.1)
Sepsis proven by positive culture	8 (4.8)	3 (4.2)	5 (5.4)	1 (3.1)	7 (5.3)	2 (5.4)	6 (4.7)	0 (0)	8 (5.2)
Noninvasive mechanical ventilation	126 (76.4)	57 (79.2)	69 (74.2)	26 (81.3)	100 (75.2)	27 (73.0)	99 (77.3)	7 (58.3)	119 (77.8)
Invasive mechanical ventilation	43 (26.1)	16 (22.2)	27 (29.0)	5 (15.6)	38 (28.6)	10 (27.0)	33 (25.8)	2 (16.7)	41 (26.8)
Prolonged use of antibiotic ^a^	34 (20.6)	21 (29.2) *	13 (14.0)	10 (31.3)	24 (18.0)	12 (32.4) *	22 (17.2)	2 (16.7)	32 (20.9)
Antibiotic treatment, mean days (95% CI)	13 (12 to 14)	14 (12 to 16)	12 (11 to 13)	13 (10 to 16)	13 (12 to 14)	14 (12 to 17)	13 (11 to 14)	14 (9 to 19)	13 (12 to 14)

Table legend: ^a^ Defined as the administration of more than 1 cycle of antibiotics during neonatal period. * *p* value < 0.05. Data are expressed as number (%), when not specified.

## Data Availability

Data are available upon reasonable request. All data relevant to the study are included in the article. Access to raw data would be provided upon request.

## References

[1] Aw M., Penn J., Gauvreau G.M., Lima H., Sehmi R. (2020). Atopic March: Collegium Internationale Allergologicum Update 2020. Int. Arch. Allergy Immunol..

[2] Sozańska B., Błaszczyk M., Pearce N., Cullinan P. (2014). Atopy and Allergic Respiratory Disease in Rural Poland before and after Accession to the European Union. J. Allergy Clin. Immunol..

[3] Grammatikos A.P. (2008). The Genetic and Environmental Basis of Atopic Diseases. Ann. Med..

[4] Groer M.W., Gregory K.E., Louis-Jacques A., Thibeau S., Walker W.A. (2015). The Very Low Birth Weight Infant Microbiome and Childhood Health. Birth Defects Res. C Embryo Today.

[5] Goedicke-Fritz S., Härtel C., Krasteva-Christ G., Kopp M.V., Meyer S., Zemlin M. (2017). Preterm Birth Affects the Risk of Developing Immune-Mediated Diseases. Front. Immunol..

[6] Cinicola B., Conti M.G., Terrin G., Sgrulletti M., Elfeky R., Carsetti R., Fernandez Salinas A., Piano Mortari E., Brindisi G., De Curtis M. (2021). The Protective Role of Maternal Immunization in Early Life. Front. Pediatr..

[7] Siltanen M., Kajosaari M., Pohjavuori M., Savilahti E. (2001). Prematurity at Birth Reduces the Long-Term Risk of Atopy. J. Allergy Clin. Immunol..

[8] Paller A.S., Spergel J.M., Mina-Osorio P., Irvine A.D. (2019). The Atopic March and Atopic Multimorbidity: Many Trajectories, Many Pathways. J. Allergy Clin. Immunol..

[9] Gonçalves C., Wandalsen G., Lanza F., Goulart A.L., Solé D., Dos Santos A. (2016). Repercussions of Preterm Birth on Symptoms of Asthma, Allergic Diseases and Pulmonary Function, 6–14 Years Later. Allergol. Immunopathol..

[10] Morata-Alba J., Romero-Rubio M.T., Castillo-Corullón S., Escribano-Montaner A. (2019). Respiratory Morbidity, Atopy and Asthma at School Age in Preterm Infants Aged 32–35 Weeks. Eur. J. Pediatr..

[11] Di Mauro A., Baldassarre M.E., Brindisi G., Zicari A.M., Tarantini M., Laera N., Capozza M., Panza R., Salvatore S., Pensabene L. (2020). Hydrolyzed Protein Formula for Allergy Prevention in Preterm Infants: Follow-Up Analysis of a Randomized, Triple-Blind, Placebo-Controlled Study. Front. Pediatr..

[12] Passariello A. (2010). Diarrhea in Neonatal Intensive Care Unit. WJG.

[13] Canani R.B., Terrin G. (2011). Recent Progress in Congenital Diarrheal Disorders. Curr. Gastroenterol. Rep..

[14] Ferreira C.R., van Karnebeek C.D.M. (2019). Inborn errors of metabolism. Handbook of Clinical Neurology.

[15] Nocerino R., Paparo L., Terrin G., Pezzella V., Amoroso A., Cosenza L., Cecere G., De Marco G., Micillo M., Albano F. (2017). Cow’s Milk and Rice Fermented with Lactobacillus Paracasei CBA L74 Prevent Infectious Diseases in Children: A Randomized Controlled Trial. Clin. Nutr..

[16] Salvia G., Cascioli C.F., Ciccimarra F., Terrin G., Cucchiara S. (2001). A Case of Protein-Losing Enteropathy Caused by Intestinal Lymphangiectasia in a Preterm Infant. Pediatrics.

[17] Passariello A., Terrin G., Cecere G., Micillo M., Marco G., Di Costanzo M., Cosenza L., Leone L., Nocerino R., Berni Canani R. (2012). Randomised Clinical Trial: Efficacy of a New Synbiotic Formulation Containing Lactobacillus Paracasei B21060 plus Arabinogalactan and Xilooligosaccharides in Children with Acute Diarrhoea. Aliment. Pharmacol. Ther..

[18] Terrin G., Boscarino G., Gasparini C., Chiara M.D., Faccioli F., Onestà E., Parisi P., Spalice A., De Nardo M.C., Dito L. (2021). Energy-Enhanced Parenteral Nutrition and Neurodevelopment of Preterm Newborns: A Cohort Study. Nutrition.

[19] Terrin G., Di Chiara M., Boscarino G., Versacci P., Di Donato V., Giancotti A., Pacelli E., Faccioli F., Onestà E., Corso C. (2020). Echocardiography-Guided Management of Preterms With Patent Ductus Arteriosus Influences the Outcome: A Cohort Study. Front. Pediatr..

[20] Terrin G., Passariello A., Canani R.B., Manguso F., Paludetto R., Cascioli C. (2009). Minimal Enteral Feeding Reduces the Risk of Sepsis in Feed-Intolerant Very Low Birth Weight Newborns. Acta Paediatr..

[21] Berni Canani R., Passariello A., Buccigrossi V., Terrin G., Guarino A. (2008). The Nutritional Modulation of the Evolving Intestine. J. Clin. Gastroenterol..

[22] Boscarino G., Conti M.G., De Luca F., Di Chiara M., Deli G., Bianchi M., Favata P., Cardilli V., Di Nardo G., Parisi P. (2021). Intravenous Lipid Emulsions Affect Respiratory Outcome in Preterm Newborn: A Case-Control Study. Nutrients.

[23] Boscarino G., Conti M.G., Gasparini C., Onestà E., Faccioli F., Dito L., Regoli D., Spalice A., Parisi P., Terrin G. (2021). Neonatal Hyperglycemia Related to Parenteral Nutrition Affects Long-Term Neurodevelopment in Preterm Newborn: A Prospective Cohort Study. Nutrients.

[24] Eichenfield L.F., Tom W.L., Chamlin S.L., Feldman S.R., Hanifin J.M., Simpson E.L., Berger T.G., Bergman J.N., Cohen D.E., Cooper K.D. (2014). Guidelines of Care for the Management of Atopic Dermatitis. J. Am. Acad. Dermatol..

[25] Brand P.L.P., Baraldi E., Bisgaard H., Boner A.L., Castro-Rodriguez J.A., Custovic A., de Blic J., de Jongste J.C., Eber E., Everard M.L. (2008). Definition, Assessment and Treatment of Wheezing Disorders in Preschool Children: An Evidence-Based Approach. Eur. Respir. J..

[26] Berni Canani R., Nocerino R., Terrin G., Di Costanzo M., Cosenza L., Troncone R. (2012). Food Allergy Diagnostic Practice in Italian Children. J. Allergy Clin. Immunol..

[27] Muraro A., Werfel T., Hoffmann-Sommergruber K., Roberts G., Beyer K., Bindslev-Jensen C., Cardona V., Dubois A., duToit G., Eigenmann P. (2014). EAACI Food Allergy and Anaphylaxis Guidelines: Diagnosis and Management of Food Allergy. Allergy.

[28] Siltanen M., Wehkalampi K., Hovi P., Eriksson J.G., Strang-Karlsson S., Järvenpää A.-L., Andersson S., Kajantie E. (2011). Preterm Birth Reduces the Incidence of Atopy in Adulthood. J. Allergy Clin. Immunol..

[29] Bernstein J.A., Lang D.M., Khan D.A., Craig T., Dreyfus D., Hsieh F., Sheikh J., Weldon D., Zuraw B., Bernstein D.I. (2014). The Diagnosis and Management of Acute and Chronic Urticaria: 2014 Update. J. Allergy Clin. Immunol..

[30] Ünal S., Kaya A., Bilgin L., Misirlioğlu E., Kocabaş C.N. (2017). Wheezing, Asthma, and Atopy in Premature Infants at 2 Years of Age. Turk. J. Med. Sci..

[31] Carstens L.E., Westerbeek E.A.M., van Zwol A., van Elburg R.M. (2016). Neonatal Antibiotics in Preterm Infants and Allergic Disorders Later in Life. Pediatr. Allergy Immunol..

[32] Morgan J., Williams P., Norris F., Williams C.M., Larkin M., Hampton S. (2004). Eczema and Early Solid Feeding in Preterm Infants. Arch. Dis. Child..

[33] Kvenshagen B., Jacobsen M., Halvorsen R. (2009). Atopic Dermatitis in Premature and Term Children. Arch. Dis. Child..

[34] Haataja P., Korhonen P., Ojala R., Hirvonen M., Paassilta M., Gissler M., Luukkaala T., Tammela O. (2016). Asthma and Atopic Dermatitis in Children Born Moderately and Late Preterm. Eur. J. Pediatr..

[35] Barbarot S., Gras-Leguen C., Colas H., Garrot E., Darmaun D., Larroque B., Roze J.C., Ancel P.Y. (2013). Lower Risk of Atopic Dermatitis among Infants Born Extremely Preterm Compared with Higher Gestational Age. Br. J. Dermatol..

[36] Trønnes H., Wilcox A.J., Lie R.T., Markestad T., Moster D. (2013). The Association of Preterm Birth with Severe Asthma and Atopic Dermatitis: A National Cohort Study. Pediatr. Allergy Immunol..

[37] Li Z., Yu M., Wang P., Qian H., Fan Y., Li X., Xu Q., Wang X., Wang X., Lu C. (2021). Association between Maternal Diabetes Mellitus and Allergic Diseases in Children—a Systematic Review and Meta-Analysis. Pediatr. Allergy Immunol..

[38] Kumar R., Ouyang F., Story R.E., Pongracic J.A., Hong X., Wang G., Pearson C., Ortiz K., Bauchner H., Wang X. (2009). Gestational Diabetes, Atopic Dermatitis, and Allergen Sensitization in Early Childhood. J. Allergy Clin. Immunol..

[39] Du Toit G., Roberts G., Sayre P.H., Plaut M., Bahnson H.T., Mitchell H., Radulovic S., Chan S., Fox A., Turcanu V. (2013). Identifying Infants at High Risk of Peanut Allergy: The Learning Early About Peanut Allergy (LEAP) Screening Study. J. Allergy Clin. Immunol..

[40] Vu K., Lou W., Tun H.M., Konya T.B., Morales-Lizcano N., Chari R.S., Field C.J., Guttman D.S., Mandal R., Wishart D.S. (2021). From Birth to Overweight and Atopic Disease: Multiple and Common Pathways of the Infant Gut Microbiome. Gastroenterology.

[41] Azad M.B., Konya T., Guttman D.S., Field C.J., Sears M.R., HayGlass K.T., Mandhane P.J., Turvey S.E., Subbarao P., Becker A.B. (2015). Infant Gut Microbiota and Food Sensitization: Associations in the First Year of Life. Clin. Exp. Allergy.

[42] Lewandowski K.C., Stojanovic N., Press M., Tuck S.M., Szosland K., Bienkiewicz M., Vatish M., Lewinski A., Prelevic G.M., Randeva H.S. (2007). Elevated Serum Levels of Visfatin in Gestational Diabetes: A Comparative Study across Various Degrees of Glucose Tolerance. Diabetologia.

[43] Gao X., Yang H., Zhao Y. (2008). Variations of Tumor Necrosis Factor-Alpha, Leptin and Adiponectin in Mid-Trimester of Gestational Diabetes Mellitus. Chin. Med. J..

[44] McGowan E.C., Bloomberg G.R., Gergen P.J., Visness C.M., Jaffee K.F., Sandel M., O’Connor G., Kattan M., Gern J., Wood R.A. (2015). Influence of Early-Life Exposures on Food Sensitization and Food Allergy in an Inner-City Birth Cohort. J. Allergy Clin. Immunol..

[45] Zutavern A., Brockow I., Schaaf B., Bolte G., von Berg A., Diez U., Borte M., Herbarth O., Wichmann H.-E., Heinrich J. (2006). Timing of Solid Food Introduction in Relation to Atopic Dermatitis and Atopic Sensitization: Results from a Prospective Birth Cohort Study. Pediatrics.

[46] Prescott S.L., Smith P., Tang M., Palmer D.J., Sinn J., Huntley S.J., Cormack B., Heine R.G., Gibson R.A., Makrides M. (2008). The Importance of Early Complementary Feeding in the Development of Oral Tolerance: Concerns and Controversies. Pediatr. Allergy Immunol..

[47] Davidson W.F., Leung D.Y.M., Beck L.A., Berin C.M., Boguniewicz M., Busse W.W., Chatila T.A., Geha R.S., Gern J.E., Guttman-Yassky E. (2019). Report from the National Institute of Allergy and Infectious Diseases Workshop on “Atopic Dermatitis and the Atopic March: Mechanisms and Interventions”. J. Allergy Clin. Immunol..

[48] Tran M.M., Lefebvre D.L., Dharma C., Dai D., Lou W.Y.W., Subbarao P., Becker A.B., Mandhane P.J., Turvey S.E., Sears M.R. (2018). Predicting the Atopic March: Results from the Canadian Healthy Infant Longitudinal Development Study. J. Allergy Clin. Immunol..

[49] Gough H., Grabenhenrich L., Reich A., Eckers N., Nitsche O., Schramm D., Beschorner J., Hoffmann U., Schuster A., Bauer C. (2015). Allergic Multimorbidity of Asthma, Rhinitis and Eczema over 20 Years in the German Birth Cohort MAS. Pediatr. Allergy Immunol..

[50] Roduit C., Frei R., Depner M., Karvonen A.M., Renz H., Braun-Fahrländer C., Schmausser-Hechfellner E., Pekkanen J., Riedler J., Dalphin J.-C. (2017). Phenotypes of Atopic Dermatitis Depending on the Timing of Onset and Progression in Childhood. JAMA Pediatr..

[51] Martinez F.D., Wright A.L., Taussig L.M., Holberg C.J., Halonen M., Morgan W.J. (1995). Asthma and Wheezing in the First Six Years of Life. N. Engl. J. Med..

[52] Lowe A.J., Angelica B., Su J., Lodge C.J., Hill D.J., Erbas B., Bennett C.M., Gurrin L.C., Axelrad C., Abramson M.J. (2017). Age at Onset and Persistence of Eczema Are Related to Subsequent Risk of Asthma and Hay Fever from Birth to 18 Years of Age. Pediatr. Allergy Immunol..

[53] Barberio G., Pajno G.B., Vita D., Caminiti L., Canonica G.W., Passalacqua G. (2008). Does a ‘Reverse’ Atopic March Exist?. Allergy.

[54] Kuo C.-H., Kuo H.-F., Huang C.-H., Yang S.-N., Lee M.-S., Hung C.-H. (2013). Early Life Exposure to Antibiotics and the Risk of Childhood Allergic Diseases: An Update from the Perspective of the Hygiene Hypothesis. J. Microbiol. Immunol. Infect..

